# WDR36 Safeguards Self-Renewal and Pluripotency of Human Extended Pluripotent Stem Cells

**DOI:** 10.3389/fgene.2022.905395

**Published:** 2022-07-22

**Authors:** Shiyu An, Dan Yao, Wenyi Zhang, Hao Sun, Tianyi Yu, Ruizhe Jia, Yang Yang

**Affiliations:** ^1^ State Key Laboratory of Reproductive Medicine, Nanjing Medical University, Nanjing, China; ^2^ Department of Obstetrics, Women’s Hospital of Nanjing Medical University, Nanjing Maternity and Child Health Care Institute, Nanjing, China; ^3^ Fourth Clinical Medicine College, Nanjing Medical University, Nanjing, China; ^4^ Department of Gynecology and Obstetrics, Affiliated Zhongda Hospital, Medical School, Southeast University, Nanjing, China

**Keywords:** self-renewal, differentiation, WDR36, p53, hEPS cells

## Abstract

Extended pluripotent stem cells (EPS cells) have unlimited self-renewal ability and the potential to differentiate into mesodermal, ectodermal, and endodermal cells. Notably, in addition to developing the embryonic (Em) lineages, it can also make an effective contribution to extraembryonic (ExEm) lineages both *in vitro* and *in vivo*. However, multiple mysteries still remain about the underlying molecular mechanism of EPS cells’ maintenance and developmental potential. WDR36 (WD Repeat Domain 36), a protein of 105 kDa with 14 WD40 repeats, which may fold into two β-propellers, participates in 18sRNA synthesis and P53 stress response. Though WDR36 safeguards mouse early embryonic development, that is, homozygous knockout of WDR36 can result in embryonic lethality, what role does WDR36 plays in self-renewal and differentiation developmental potential of human EPS cells is still a subject of concern. Here, our findings suggested that the expression of WDR36 was downregulated during human hEPS cells lost self-renewal. Through constructing inducible knockdown or overexpressing WDR36-human EPS cell lines, we found that WDR36 knockdown disrupted self-renewal but promoted the mesodermal differentiation of human EPS cells; however, overexpressing of WDR36 had little effect. Additionally, P53 inhibition could reverse the effects of WDR36 knockdown, on both self-renewal maintenance and differentiation potential of human EPS cells. These data implied that WDR36 safeguards self-renewal and pluripotency of human EPS cells, which would extend our understanding of the molecular mechanisms of human EPS cells’ self-renewal and differentiation.

## Introduction

Pluripotent stem cells (PSCs), including embryonic stem cells (ESCs) and induced pluripotent stem cells (iPSCs), have an unlimited self-renewal capacity and the potential to differentiate into cell types representing the three embryonic germ layers—mesoderm, ectoderm, and endoderm (Ying et al., 2008). Due to these properties, PSCs, an excellent system for modeling embryonic development, occurrence, and development of disease, may contribute to the development of cell-replacement therapies.

Previous work has shown that the self-renewal of PSCs is coordinated by multiple transcription factors and RNA regulators (Huang et al., 2015; Abu-Dawud et al., 2018). Mouse, human, and rat PSCs are regulated by a common subset of transcription factors for “stemness,” among which OCT4, SOX2, and NANOG are constitutive core pluripotency factors (Boyer et al., 2005). OCT4-deficient embryos are viable at the morula stage but cannot form the intact inner cell mass (ICM) *in vivo* and ESC colony *in vitro*, suggesting that OCT4 plays an important role in the maintenance of ESCs (Nichols et al., 1998). SOX2 is critical for PSC self-renewal and pluripotency, and it has been shown that knockdown or conditional deletion of SOX2 leads to trophoblast differentiation (Masui et al., 2007). NANOG is a protein containing a homologous structural domain that interacts with SOX2 and OCT4 to establish the stemness of PSCs. Overexpression of NANOG in mouse PSCs will promote self-renewal and stabilize the undifferentiated state by establishing endogenic self-renewal, which is independent of growth factors or small molecules (Chambers et al., 2003), whereas in human PSCs it allows feeder-free propagation for multiple passages (Darr et al., 2006). Recent studies have identified many additional transcription factors in the regulatory network of PSCs. Importantly, many of these self-renewal-related factors work together to maintain pluripotency. These factors also act as hubs between external signaling pathways and internal determinants of pluripotency (Huang et al., 2015). OCT4, SOX2, and NANOG also occupy repressed genes which encode cell lineage-specific regulators, and the strict control of these genes is essential for PSCs to maintain a stable pluripotent state or to undergo normal differentiation. For example, the precise expression level of OCT4 regulates three distinct fates of PSCs: a less than two-fold increase in OCT4 expression results in differentiation toward primitive endoderm and mesoderm. In contrast, the repression of OCT4 induces loss of pluripotency and commitment to trophectoderm (Niwa et al., 2000). Furthermore, a slowdown of the cell cycle also aids differentiation (Li and Kirschner, 2014), indicating an integral role of the cell cycle in the maintenance of pluripotency and differentiation.

More recently, Yang et al. (2017) have developed a culture condition that endowed canonical human PSCs with totipotent-like features, thereafter named extended pluripotent stem (hEPS) cells, which can efficiently contribute to both embryonic (Em) and extraembryonic (ExEm) lineages not only *in vitro* but also *in vivo*. Most notably, some laboratories have successfully generated blastocyst-like structures (EPS-blastoids), which resemble blastocysts in morphology and cell-lineage allocation and recapitulate key morphogenetic events during pre-implantation and early post-implantation development *in vitro* (Fan et al., 2021; Sozen et al., 2021), by using human EPS through lineage segregation and self-organization. However, little is known about the underlying molecular mechanism of EPS maintenance and developmental potential.

The WD40 repeat (WDR) domain is one of the most abundant protein interaction domains in the human proteome, with over 360 domains annotated to date. These structural domains are important subunits of multiprotein complexes involved in multiple signaling pathways, such as DNA damage sensing and repair, epigenetic regulation of gene expression and chromatin organization, ubiquitin signaling and protein degradation, cell cycle, and immune-related pathways (Schapira et al., 2017). WDR36, a 105-kDa protein with 14 WD40 repeats which may fold into two β-propellers (Footz et al., 2009), is expressed in multiple tissues, with significant mRNA organization in the heart, skeletal muscle, pancreas, liver, and placenta (Monemi et al., 2005). The primary structure of WDR36 protein is similar to that of Utp21, a nucleolar ribonucleoprotein (Kressler et al., 2010), which might explain why the depletion of WDR36 in human cells leads to delay of 18S rRNA processing, disruption of nucleolar morphology, and activation of the P53 stress response pathway (Gallenberger et al., 2011). The tumor suppressor gene *P53* is known to be described as the “guardian of the genome” because it can protect cells from tumor transformation. In addition to being involved in the regulation of DNA repair, apoptosis, and senescence, it also supervises processes such as self-renewal and differentiation of stem cells (Lin et al., 2005; Qin et al., 2007; Chen, 2016; Koifman et al., 2019), as well as iPSC reprogramming (Marion et al., 2009; Utikal et al., 2009). WDR5, another member having the WD40 repeat domain, was confirmed to regulate P53 stability and directly interact with P53 during ESC specification. It could also interact with OCT4, CTCF, or lncRNA to facilitate iPSC reprogramming and maintain ESC identity (Ang et al., 2011; Li et al., 2020).

Accordingly, we wonder whether WDR36 plays a pivotal role in the self-renewal and differentiation potential of hEPS cells. Through constructing inducible knockdown or overexpressing WDR36-hEPS cell lines, we found that WDR36 regulated the self-renewal of hEPS cells, providing a new regulatory target. Moreover, we determined that WDR36-knockdown promoted the differentiation of human EPS cells, while overexpressing of WDR36 had little effect. Additionally, P53 inhibition could reverse the effects of WDR36 knockdown on hEPS cells.

## Materials and Methods

### Cell Culture

Human EPS cells (hEPS1 and hEPS2, two cell lines of hEPS) were cultured in the serum-free N2B27-LCDMY medium under 20% O_2_ and 5% CO_2_ at 37°C. The N2B27 medium was prepared as follows: 1:1 mixture of DMEM/F12 (Thermo Fisher Scientific, 11330-032) and Neurobasal (Thermo Fisher Scientific, 21103-049), 0.5X N2 supplement (Thermo Fisher Scientific, 17502-048), 0.5X B27 supplement (Thermo Fisher Scientific, 12587-010), 1% nonessential amino acids (Thermo Fisher Scientific, 11140-050), 1% GlutaMAX™ (Thermo Fisher Scientific, 35050-061), 0.1 mM β-mercaptoethanol (Thermo Fisher Scientific, 21985-023), and 1% penicillin–streptomycin (Thermo Fisher Scientific, 15140-122). To prepare the N2B27-LCDMY medium, small molecules and cytokines were added to the N2B27 medium as follows: 10 ng/ml recombinant human LIF (L, 10 ng/ml; StemImmune, EST-LIF-0100), CHIR 99021 (C, 1 μM; MCE, HY-10182), (S)-(+)-dimethindene maleate (D, 2 μM; Tocris, 1425), minocycline hydrochloride (M, 2 μM; Santa Cruz Biotechnology, sc-203339), and Y-27632 (2 μM; Hanxiang, 17109). Human EPS cells were cultured on mitomycin C (Sigma-Aldrich, M4287)-inactivated mouse embryonic fibroblast (MEF) feeder cells (3 × 10^4^ cells per cm^2^). The N2B27-LCDMY medium was changed daily. Human EPS cells were passaged by single-cell trypsin digestion (0.05% trypsin-EDTA, Thermo Fisher Scientific, 25300-062) every 3 days (normally at a split ratio of 1:3–1:6).

### Establishment of Doxycycline-Inducible WDR36-Modified hEPS Cell Lines

We first generated the WDR36 short hairpin RNA (shRNA) vector from a PiggyBac TetR puro vector by inserting multiple cloning sites (MCSs) and further cloned human WDR36 shRNA oligonucleotides into MCS (EcoR I and Age I sites), and then these plasmids were confirmed by sequencing. The mutually priming oligonucleotides used in this study are listed in Supplementary Table S3. The WDR36 overexpression vector was produced from the PiggyBac TetOn3G tdTomato puro vector by inserting multiple cloning sites (MCSs) and further cloned human WDR36 cDNA into MCS (EcoR I and Age I sites) to generate the TRE3G-WDR36-T2A-tdTomato plasmid.

By using the Lipofectamine Stem Transfection Reagent (Thermo Scientific, STEM00001) according to the manufacturer’s instruction, the WDR36 shRNA and pCyL43 (PiggyBac transposase) vectors were co-transfected into hEPS1 and hEPS2 cells separately to make the inducible shWDR36-hEPS cell lines. To generate the inducible overexpression WDR36-hEPS cell line, TRE3G-WDR36-T2A-tdTomato and pCyL43 vectors were co-transfected into hEPS1 cells. Puromycin (0.25 μg/ml) was added for positive clone selection for 7 days, and the expanded clones were designated as the overexpressed or knockdown hEPS cells, respectively. The gene expression level of WDR36 was verified by quantitative RT-PCR (qRT-PCR) and Western blot analysis.

### Immunofluorescence Staining

Cells were fixed with 4% paraformaldehyde for 20 min at room temperature (RT) and then permeabilized and blocked with PBS containing (vol/vol) 0.25% Triton X-100 (Beyotime, P0096) together with (vol/vol) 2.5% donkey serum (Jackson ImmunoResearch, 017-000-121) for 50 min at RT. The primary antibody (information about antibodies is listed in Supplementary Table S1) with the blocking solution was diluted, and the sample was incubated for at least 12 h at 4°C. Subsequently, the samples were washed five times with PBS for 3 min each and then incubated at RT with the secondary antibodies diluted in 2.5% donkey serum (information about antibodies is listed in Supplementary Table S1). After 1 h, the samples were washed three times with PBS, and the nuclei were stained with 4′,6-diamidino-2-phenylindole (DAPI) (YIFEIXUE BIO TECH, YD0020-10). We observed the results using confocal microscopy (ZEISS LSM700, Germany).

### RNA Isolation and qRT-PCR Analysis

Total RNA was extracted from cells using the TRIzol Reagent (Invitrogen 15596-026) as described by the manufacturer’s instructions, and then RNA was reverse-transcribed to cDNA using a PrimeScript™ RT reagent kit with gDNA Eraser (Takara, Dalian, China). To analyze the relative expression levels of genes in the cultured hEPS1 and hEPS2 cells, the qRT-PCR was performed in a Step One Plus Real-Time PCR System (Applied Biosystems, CA, United States), using the FastStart Universal SYBR Green Master kit (Vazyme, Nanjing, China). The primers were designed with Primer 5 software and are listed in Supplementary Table S2. The melting curve of each mRNA was used to evaluate the amplification quality. The expression data were assessed by the 2^−△△CT^ method, and the expression level of glyceraldehyde 3-phosphate dehydrogenase (*GAPDH*) was used as an endogenous normalization control.

### Western Blot Analysis

Total protein was extracted from the cultured hEPS1 and hEPS2 cells with the addition of RIPA buffer (Beyotime, Beijing, China) containing 1 mM phenylmethylsulfonyl fluoride and quantified *via* the BCA method. The protein samples were boiled in water for 10 min and then 30 µg of total protein was electrophoresed for each sample in 8% SDS-polyacrylamide gels, followed by being transferred onto polyvinylidene difluoride membranes (Millipore, MA, United States). After being blocked with 5% non-fat dried milk for 2 h, we incubated the membranes overnight with primary antibodies at 4°C. Then, they were incubated with the secondary antibodies for 1 h at RT. Details of the antibodies are provided in Supplementary Table S1. After being washed three times, bands were detected with the enhanced chemiluminescence (ECL) detection kit (TransGen Biotech, Beijing, China). We used ImageJ software (Wayne Rasband, MD, United States) to calculate grayscale values, and GAPDH played as a reference. The experiments were repeated three times.

### Embryoid Body (EB) Formation Assay

The embryoid body (EB) formation assay was based on the previous study by Liu et al. (2021). hEPS1 cells were rinsed once and digested with 0.05% trypsin-EDTA (Thermo Fisher Scientific, 25300-062) for 3 min in a 37°C incubator. Then, the cell suspension was collected and centrifuged at 900 rpm for 4 min. After being rinsed with DMEM/F12 and centrifugated, the supernatant was removed, and the colonies were resuspended in the EB medium (DMEM/F12 containing 20% KSR (knockdown serum replacement), 1% GlutaMAX™, 1% nonessential amino acids, and 55 μM β-mercaptoethanol and cultured in low-adherent cell culture dishes. Y27632 was also added into the EB medium during the first 24 h to ensure high cell viability. The EB medium was changed daily and EBs samples were collected on days 2, 4, and 6 for qRT-PCR analysis. For plating, the cell clumps were transferred into DMEM/F12 containing 20% FBS on the gelatin-coated 24-well plate for 5–7 days and fixed for further analysis.

### Committed Mesodermal Differentiation

Committed mesodermal differentiation was performed according to the reported protocol by Lian et al. (2013). Approximately 30% confluent of hEPS1 and hEPS2 cells were treated with 5 µM Y-27632 (Hanxiang, 17109) and 6 µM CHIR99021 (MCE, HY-10182) in the mesoderm differentiation induction media containing RPMI1640, 100X B27 (Thermo Fisher Scientific, A1895601) for 4 days. Later, the differentiated cells were fixed and stained for markers of the mesodermal germ layer.

### Statistical Analysis

All data were presented as mean values ±standard error of the mean (SEM). Statistical analysis was performed using the two-tailed Student’s *t*-test or one-way analysis of variance (ANOVA) and was defined as statistically significant when *p* < 0.05.

## Results

### WDR36 Expression Was Downregulated During hEPS Cell Differentiation

**FIGURE 1:**
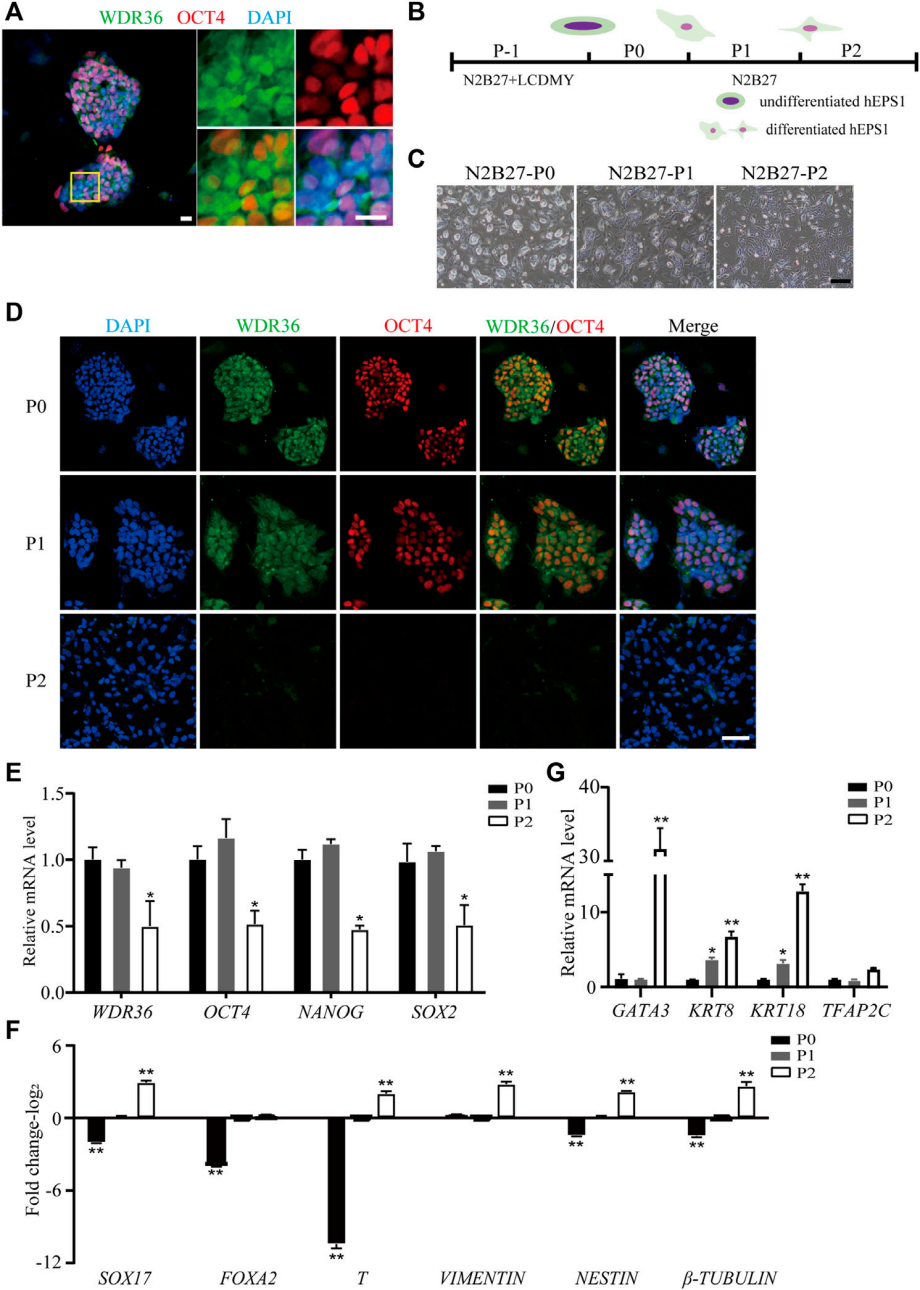
Down-expression of WDR36 during hEPS1 cell differentiation. **(A)** Expression of WDR36 protein in hEPS1 cells. Bars = 20 μm. **(B)** Flow chart of hEPS1 cells in a spontaneous differentiation model. **(C,D)** Representative brightfield [**(C)**, Bar = 200 μm] and immunofluorescent images [**(D)**, Bar = 50 μm] of hEPS1 cells in the spontaneous differentiation assay at P0, P1, and P2, separately. **(E–G)** Expression of pluripotent genes **(E)**, embryonic germ layer-related genes **(F),** and extraembryonic differentiation-related genes **(G)** in hEPS1 cells cultured in N2B27 medium at P0, P1, and P2. *n* = 3 experiments; mean ± S. D; two-tailed Student’s *t*-test. *, 0.01 < *p* < 0.05; **, *p* < 0.01; no labeling indicates no statistical significance.

To understand the role of WDR36 in hEPS cells’ maintenance, we first detected the expression of WDR36 in hEPS1 and hEPS2 cells cultured in the N2B27-LCDMY medium (undifferentiated state). We found that WDR36 is mainly distributed both in the cytoplasm and nucleus, while a few are only in the nucleus of cells (Figure 1A and Supplementary Figure S1A). When LCDMY was withdrawn, hEPS1 cells initiated to differentiate, losing their dome-shaped clone morphology gradually (Figures 1B,C), and WDR36 tended to locate mainly in the cytoplasm of cells, especially at passage 1 (Figure 1D). After being exposed to the condition without LCDMY cocktail for two passages, all the hEPS1 cells lost their EPS identity with no OCT4 or WDR36 expression (Figure 1D). To be similar but slightly different, hEPS2 differentiated more quickly than hEPS1, completely losing self-renewal after passage 1 (Supplementary Figures S1B,C). All the hEPS2 cells expressed WDR36 only in their cytoplasm as early as LCDMY being removed for 3 and 4 days (passage 0) and were OCT4- and WDR36-negative at passage 1 (Supplementary Figures S1C). Furthermore, data indicated that the expressions of *WDR36*, *NANOG*, *OCT4,* and *SOX2* were decreased during the differentiated process of hEPS1 and hEPS2 at transcriptional levels (Figure 1E and Supplementary Figures S1D), while the expression of three embryonic germ layer markers—endoderm (*SOX17* and *FOXA2*), mesoderm (*T* and *vimentin*), and ectoderm (*nestin* and *β-tubulin*)—increased (Figure 1F and Supplementary Figures S1E). Considering hEPS can efficiently contribute to both embryonic and extraembryonic lineages both *in vitro* and *in vivo*, we also detected the expression of extraembryonic differentiation-related genes. The results showed that *GATA3*, *KRT8*, *KRT18*, and *TFAP2C* were significantly increased during hEPS1 and hEPS2 differentiation (Figure 1G and Supplementary Figures S1F). The aforementioned results showed that the WDR36 expression was downregulated during hEPS cell differentiation.

### Inhibition of WDR36 Impaired hEPS Cells’ Self-Renewal

**FIGURE 2:**
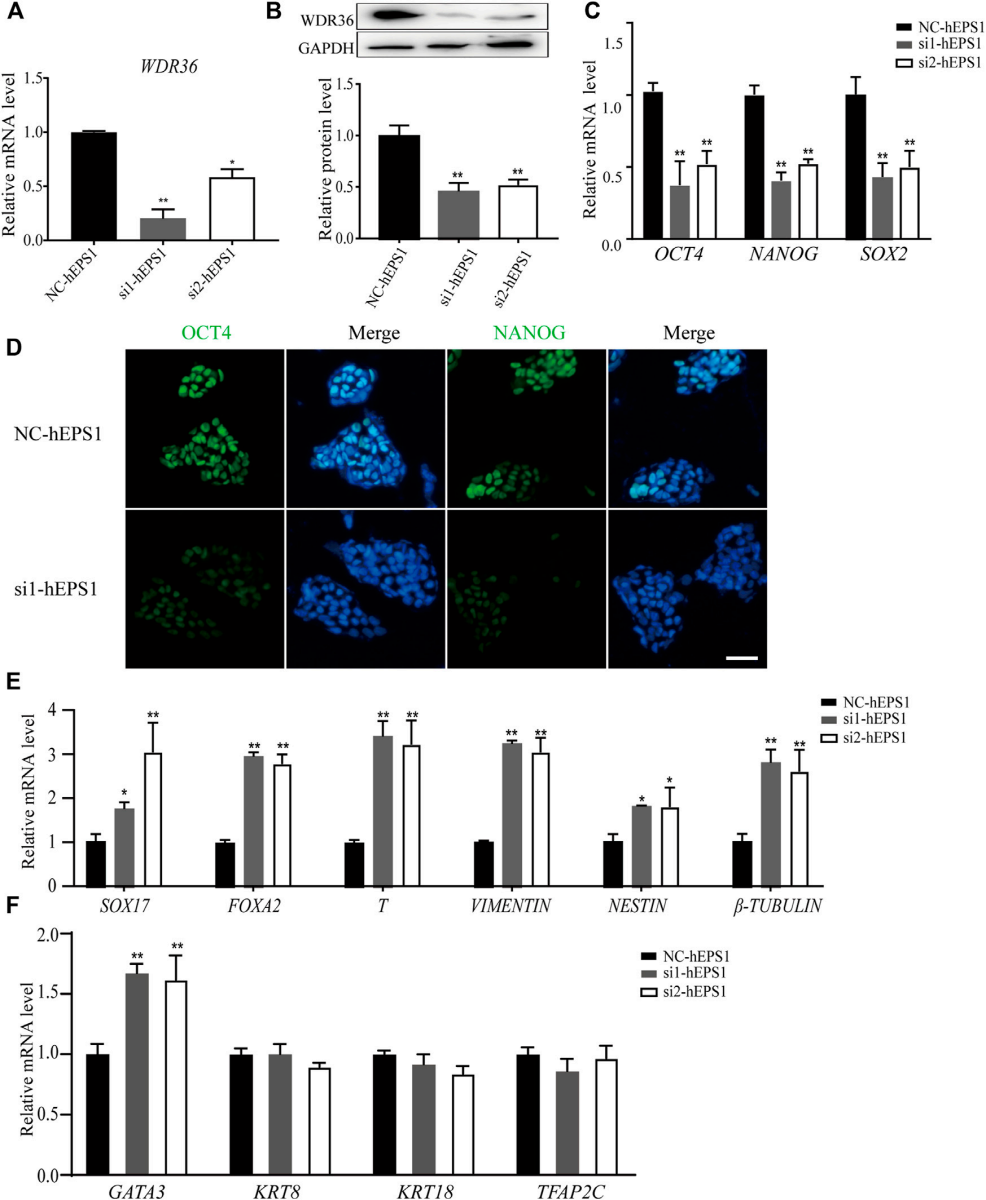
siRNA-WDR36 would impair self-renewal of hEPS1 cells. **(A,B)** WDR36 expression level in hEPS1 cells which had been transfected with WDR36 siRNA was determined using qRT-PCR **(A)** and western blotting **(B)**. **(C)** Expression of *OCT4*, *NANOG* and *SOX2* mRNA in hEPS1 cells transfected with WDR36 siRNA. **(D)** Immunofluorescent images of *OCT4* and *NANOG* in hEPS1 cells with WDR36 siRNA. Bar = 50 μm. **(E,F)** Expression of three embryonic germ layer related genes **(E)** and extraembryonic differentiation related genes **(F)** in siRNA-WDR36 hEPS1 cells. *n* = 3 experiments; mean ± S.D; two-tailed Student’s t-test. *, 0.01 < *p* < 0.05; **, *p* < 0.01; no labeling indicates no statistical significance. NC-hEPS1: hEPS1 transfected with siRNA-scramble; si1-hEPS1 and si2-hEPS1: hEPS1 transfected with small interference RNA 1 or RNA 2 of WDR36 respectively.

Mechanistic studies have identified WDR36 as a functional homolog of yeast Utp21 (Skarie and Link, 2008), which is related to 18S rRNA maturation, and rRNA synthesis plays a role in self-renewal regulation in PSCs. We hypothesized that WDR36 played an impotent role in hEPS cells’ self-renewal. The specific small-interference RNAs (siRNAs) for WDR36 (NCBI accession number: NM_139281.2), and siRNA-control (Supplementary Table S3) was synthesized and purified at Tsingke (Nanjing, China), and the most efficient two siRNAs were selected as candidates and further confirmed by qRT-PCR and Western blotting experiments (Figures 2A,B, and Supplementary Figure S2A). We noticed robustly reduced expression of *OCT4*, *NANOG*, and *SOX2* in siRNA-WDR36 hEPS1 cells compared with siRNA-control (Figure 2C), consistent with the results of immunofluorescence (Figure 2D). This assay was performed by using hEPS2, and a similar phenomenon was also observed (Supplementary Figure S2B). Moreover, as shown in Figure 2E, embryonic germ layer markers (*SOX17*, *FOXA2*, *T*, *vimentin*, *nestin*, and *β-tubulin*) all significantly enhanced in the siRNA-WDR36 groups. In addition, we found that WDR36 inhibition induced an increase in *GATA3* expression, while the expressions of *KRT8*, *KRT18*, and *TFAP2C* did not undergo pronounced changes (Figure 2F). Collectively, our findings suggested that WDR36 impaired the self-renewal of hEPS.

### Dox-Induced WDR36 Knockdown Elevated Mesodermal Differentiation Potential of hEPS Cells *In Vitro*

To further explore what role WDR36 played in differentiation potency of hEPS, we constructed two Dox-induced WDR36 knockdown sub-cell lines based on hEPS1 (Supplementary Figure S3A), namely, sh1-hEPS1 and sh2-hEPS1. After adding Dox, qRT-PCR and protein blot analysis confirmed successful construction; thus, sh1-hEPS1 was selected for subsequent experiments due to the higher silencing efficiency (Supplementary Figures S3B,C).

**FIGURE 3:**
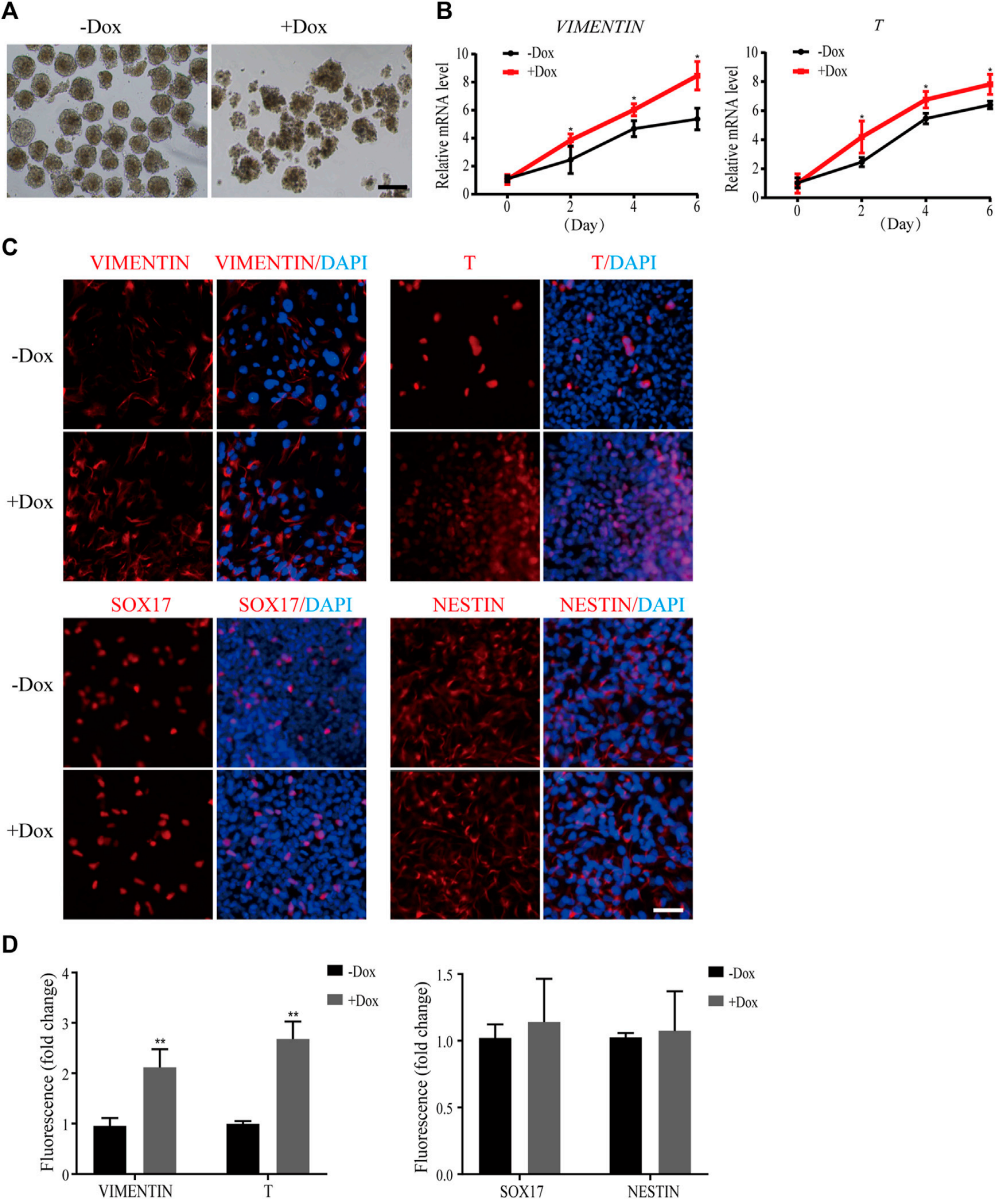
Dox-induced WDR36 knockdown elevated differentiation potential of hEPS1 cells in an EB formation assay. **(A)** Representative images of WDR36-modified hEPS1 cells forming EBs in the EB medium. On day 7, EBs were collected and observed under the microscope. Bar = 200 μm. **(B)** Expression of mesodermal genes in WDR36-modified EBs treated with or without Dox. **(C,D)** Immunofluorescent images **(C)** and quantification intensity for immunofluorescence **(D)** of WDR36-modified EBs treated with or without Dox (2 μg/ml). Bar = 50 μm; *n* = 3 experiments; mean ± S. D; two-tailed Student’s *t*-test. *, 0.01 < *p* < 0.05; **, *p* < 0.01; no labeling indicates no statistical significance.

Next, we simulated early human embryonic development by EB formation assay. As shown in Figure 3A, the morphology of EBs became irregular when WDR36 was knocked down, indicating that the spontaneous differentiation potential of hEPS1 cells was disrupted. In addition, we collected EBs on days 2, 4, and 6 for mRNA level confirmation by qRT-PCR. We observed a significant decrease in the expressions of *OCT4* and *NANOG* as spontaneous differentiation proceeded (Supplementary Figure S4A). Furthermore, the results suggested that WDR36 knockdown promoted mesodermal differentiation potential of hEPS1 cells (Figure 3B), whereas no significant change in ectodermal or endodermal differentiation was exhibited, and so did the expressions of extraembryonic specific genes *GATA3* and *KRT8* (Supplementary Figure S4A). Then, EBs adhered to Matrigel for 5–7 days for further analysis, and the immunofluorescent results also showed a similar tendency, in comparison to the untreated group (Figures 3C,D). The aforementioned evidence indicated that WDR36 had an important role in germ layer differentiation of hEPS1 cells.

**FIGURE 4:**
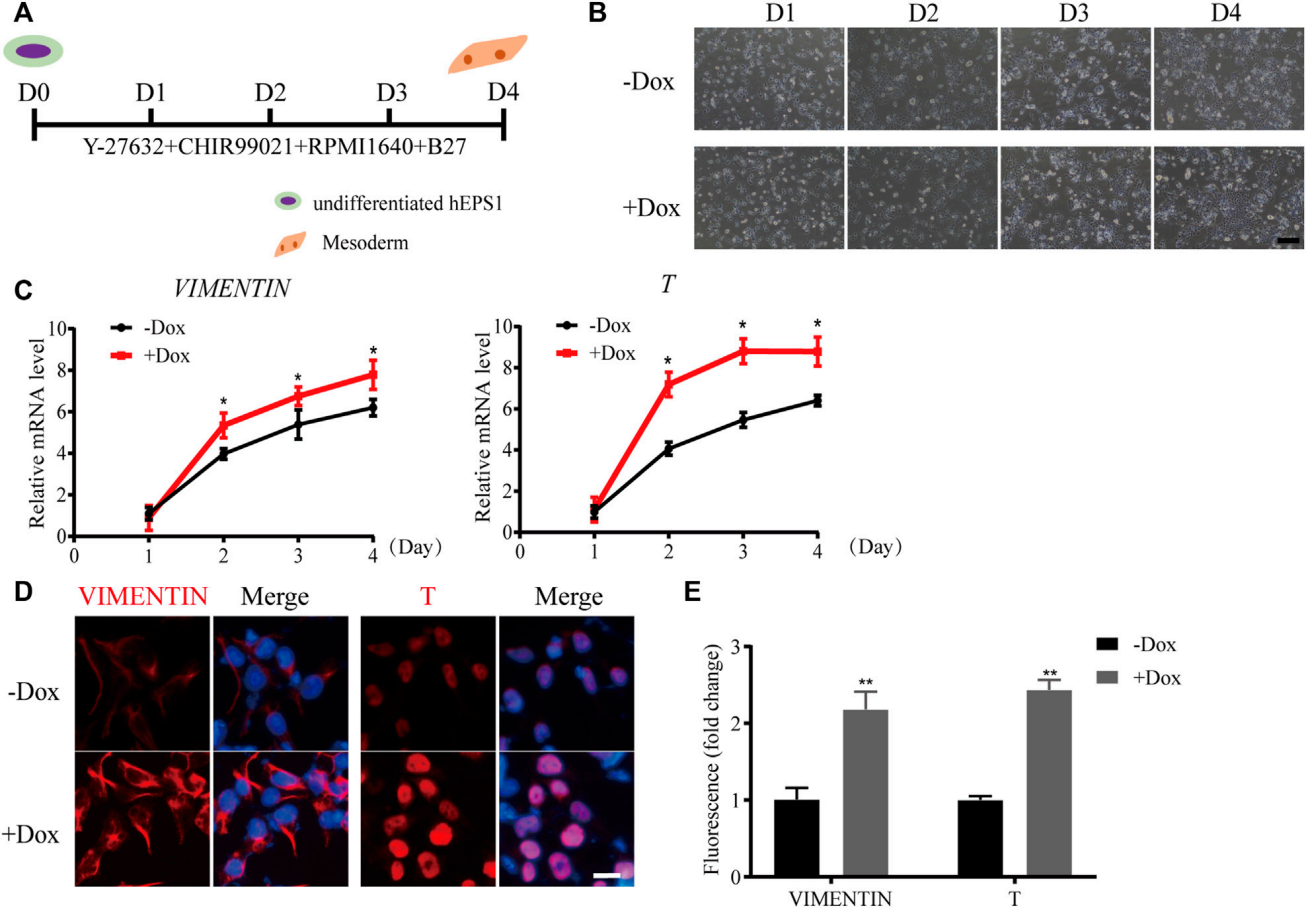
WDR36 knockdown promoted mesodermal differentiation potential of hEPS1 cells in a mesodermal-committed differentiation assay. **(A)** Time scheme of the mesodermal-committed differentiation assay. **(B)** Representative images of Dox-induced shWDR36 hEPS1 cells in mesoderm differentiation medium with or without Dox. Bar = 200 μm. **(C,D)** mRNA **(C)** and protein expression **(D)** of T and vimentin in sh1-hEPS1 cells treated with or without Dox. Bar = 50 μm. **(E)** Quantification intensity levels for immunofluorescence of T and vimentin in sh1-hEPS1 cells treated with or without Dox. *n* = 3 experiments; mean ± S. D; two-tailed Student’s *t*-test. *, 0.01 < *p* < 0.05; **, *p* < 0.01; no labeling indicates no statistical significance.

Since mesodermal lineage genes increased obviously during sh1-hEPS1 forming EBs, we hypothesized that WDR36 inhibition would promote mesodermal differentiation of hEPS. To address this, we carried out a mesodermal-committed differentiation assay (Figure 4A). We found no significant morphological difference between Dox-treated and -untreated sh1-hEPS1 cells during differentiation (Figure 4B). However, robustly higher expressions of *T* and vimentin were noted in the Dox-induced group (Figure 4C), consistent with the results of immunofluorescence (Figures 4D,E). In order to confirm this in hEPS2, we constructed a Dox-induced WDR36 knockdown sub-cell line called sh1-hEPS2 and successfully confirmed the Dox-induced inhibition effect by qRT-PCR and protein blot (Supplementary Figures S5A,B). As expected, WDR36 inhibition boosted mesodermal differentiation of hEPS2 (Supplementary Figures S5C–F).

### WDR36-Overexpression Hardly Affected Self-Renewal and Differentiation of hEPS1 Cells

In addition to Dox-induced shRNA cell lines, we also constructed a Dox-induced WDR36-overexpression hEPS1 cell line to evaluate the effect of WDR36 overexpression on hEPS self-renewal and differentiation (Supplementary Figure S6A). Following Dox treatment, both qRT-PCR and Western blot results confirmed the successful construction (Supplementary Figures S6B,C). We then asked whether WDR36-overexpression affected the self-renewal and differentiation of hEPS1. We observed slight upregulation of *OCT4* and *NANOG*, but no statistical difference was observed (Supplementary Figure S6D). Additionally, immunofluorescent results showed that there was no difference in the protein levels (Supplementary Figure S6E).

We further examined whether WDR36-overexpression would affect the differentiation potential of hEPS1 by EB formation assay. As shown in Supplementary Figure S7A, no significant difference in EB morphology was observed in Dox-induced WDR36-overexpressing hEPS1 cells. The qRT-PCR results showed that as spontaneous differentiation proceeded, pluripotent genes *OCT4* and *NANOG* decreased (Supplementary Figure S7B), while differentiated genes enhanced (Supplementary Figure S7C). During this procedure, no apparent difference was found between Dox-treated and -untreated groups (Supplementary Figures S7C,D). Thus, we indicated that neither self-renewal nor differentiation potential would be affected by overexpression of WDR36.

### P53 Inhibition Could Reverse the Effect of WDR36 Inhibition on hEPS1 Cells

**FIGURE 5:**
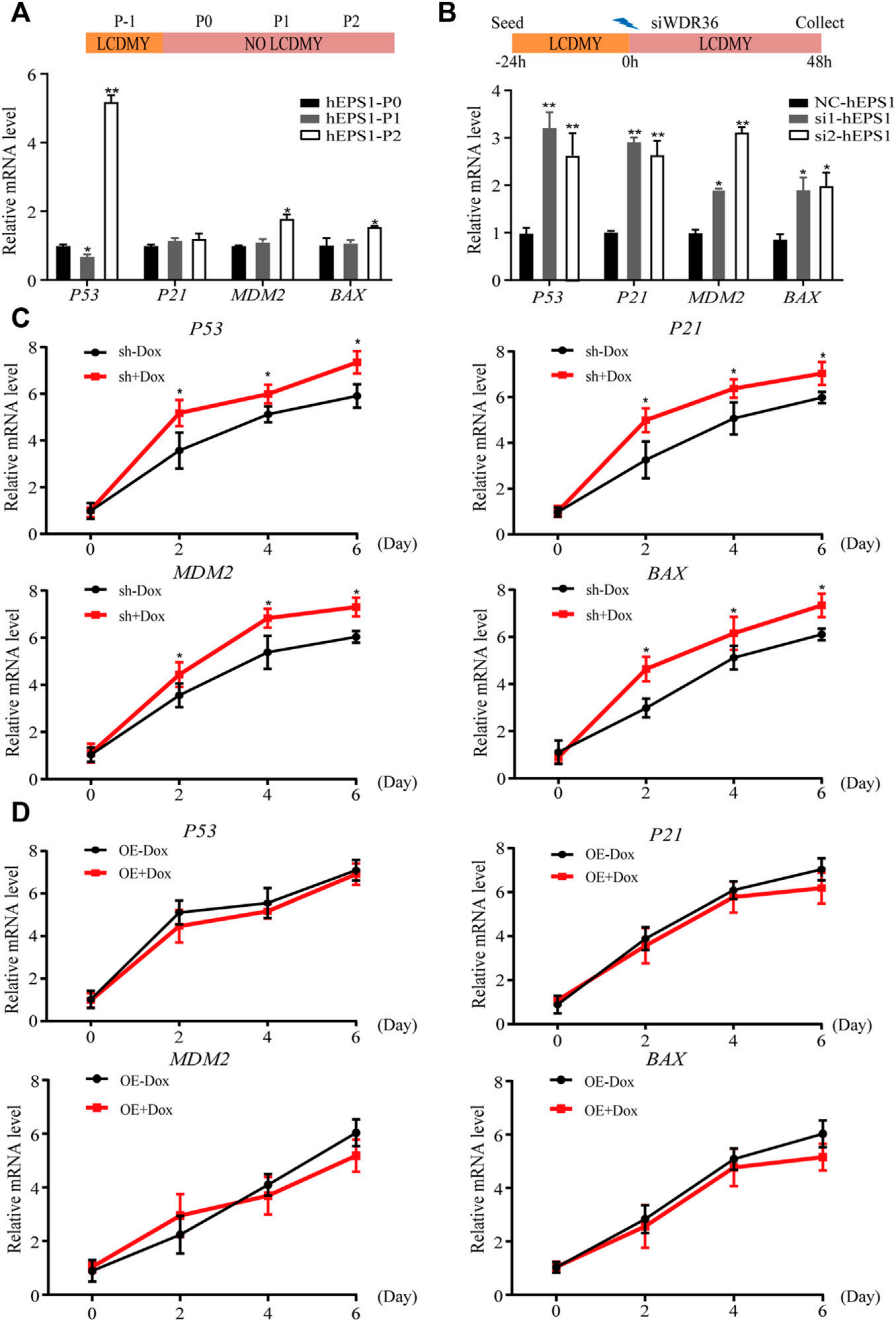
Expression of P53 pathway-related genes during the spontaneous differentiation procedure of hEPS1 cells. **(A)** Expression of *P53*, *P21*, *MDM2*, and *BAX* in hEPS1 cells which were cultured in the N2B27 medium at P0, P1, and P2. **(B)** Expression of *P53*, *P21*, *MDM2*, and *BAX* in hEPS1 cells with WDR36 siRNA. NC: siRNA-scramble; si1: small interference RNA 1 of WDR36; si2: small interference RNA 2 of WDR36. **(C)** Expressions of *P53*, *P21*, *MDM2*, and *BAX* in Dox-induced WDR36 knockdown EBs treated with or without Dox. **(D)** Expressions of *P53*, *P21*, *MDM2*, and *BAX* in Dox-induced WDR36 overexpression EBs treated with or without Dox. OE: overexpression of WDR36. *n* = 3 experiments; mean ± S. D; two-tailed Student’s t-test. *, 0.01 < *p* < 0.05; **, *p* < 0.01; no labeling indicates no statistical significance.

Multiple studies have reported that tumor suppressor P53 plays an active role in promoting differentiation and opposing self-renewal of human ESCs (Jain et al., 2012), and loss of WDR36 causes an activation of the P53 stress-response pathway (Skarie and Link, 2008). To determine whether P53 inhibition could recover the effect of WDR36 inhibition on hEPS1 cells, we first checked whether P53 had similar effects on hEPS1 cells compared to traditionally primed human PSCs. As expected, *P53* was activated when LCDMY was withdrawn from the culture condition, which resulted in differentiation. Meanwhile, the expressions of *P21*, *MDM2*, and *BAX* were also increased but not so significant (Figure 5A), which was in accordance with Lin et al. (2005). Furthermore, the expressions of *P53*, *P21*, *MDM2*, and *BAX* in siRNA-WDR36 hEPS1 cells were significantly increased compared with those of siRNA-control cells (Figure 5B). We then asked whether similar results were present during the EB differentiation. We observed a significant upward reversion in *P53* expression as expected, and the expressions of *P21*, *MDM2*, and *BAX* also showed an upward trend in WDR36-knockdown hEPS1 cells compared with those of Dox-untreated cells (Figure 5C). However, the expressions of *P53*, *P21*, *MDM2*, and *BAX* did not drop down obviously after overexpression of WDR36 (Figure 5D).

Thus, we wondered whether inhibition of the P53 pathway could recover the inhibitory effect of WDR36 knockdown by treating hEPS1 cells with pifithrin-alpha (PFT-α), a canonical inhibitor of the P53 pathway (Ulmke et al., 2021). First of all, we verified the inhibitory effect of PFT-α on the expression of P53 and P21 in hEPS1 cells at different concentrations. The qRT-PCR and Western blot results indicated that the expressions of P53 and P21 were effectively inhibited in 10 μM of PFT-α treatment and did not affect the survival of hEPS1 cells (Supplementary Figures S8A,B). Therefore, we chose 10 μM PFT-α for subsequent experiments. As shown in Supplementary Figures S8C,D, PFT-α treatment induced notable improvement in the expressions of pluripotent genes *OCT4*, *NANOG*, and *SOX2*, which was consistent with the immunofluorescent results. Furthermore, we also found that PFT-α treatment could impair the commitment of hEPS1 cells into mesodermal lineage (Supplementary Figures S8E,F). These results implied that P53 played an important role in the differentiation of hEPS1 cells.

**FIGURE 6:**
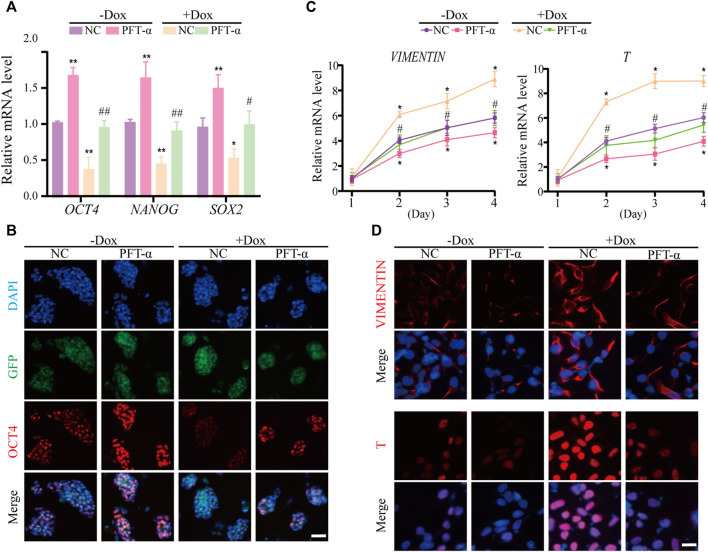
Inhibition of P53 could restore the influences of WDR36 knockdown on hEPS1 cells. **(A)** qRT-PCR analysis of the Dox-induced shWDR36 hEPS1 cells cultured under the condition with or without Dox. **(B)** Immunofluorescent images of OCT4 in Dox-induced shWDR36 hEPS1 cells. **(C)** Expression of T and vimentin in Dox-induced shWDR36 hEPS1 cells. **(D)** Immunofluorescent images of T and vimentin in Dox-induced shWDR36 hEPS1 cells. Bars = 50 μm. NC, culture condition did not contain 10 μM PFT-α. *n* = 3 experiments; mean ± S. D; one-way analysis of variance (ANOVA). *, 0.01 < *p* < 0.05; **, *p* < 0.01; #, 0.01 < *p* < 0.05; ##, *p* < 0.01; no labeling indicates no statistical significance.

Previous studies have shown that P53 inhibits NANOG expression and downregulates stemness of stem cells (Lin et al., 2005), and loss of WDR36 leads to activation of the P53 stress-response pathway (Skarie and Link, 2008), suggesting that defects in the P53 pathway may affect the regulation of hEPS1 cells by WDR36. Thus, we examined whether PFT-α could reverse the effects of WDR36 knockdown on the self-renewal and mesoderm differentiation potential of hEPS1 cells. The qRT-PCR and immunofluorescence staining revealed that WDR36 knockdown significantly inhibited the self-renewal of hEPS1 cells, while PFT-α treatment could rescue the effects (Figures 6A,B). Furthermore, we reconfirmed the effect of PFT-α in a committed mesodermal differentiation assay. The data showed that WDR36 knockdown significantly promoted the mesoderm differentiation potential of hEPS1 cells. However, when hEPS1 cells were co-treated with WDR36 shRNA and PFT-α, the mesoderm differentiation potential was attenuated (Figures 6C,D). These results showed that knocking-down WDR36 significantly regulated hEPS1 cells’ self-renewal and differentiation potential, and inhibition of P53, in turn, restored these effects.

## Discussion

PSCs are capable of self-renewal and can differentiate into different cell types under certain differentiation conditions. Their self-renewal and differentiation ability is precisely regulated by various regulatory factors at the transcriptional, translational, and post-translational levels (Liou et al., 2017). The maintenance of pluripotency, the commitment to a specific lineage fate, and the switch to cell differentiation depend on the tight regulation of protein synthesis and ribosome biogenesis (Saba et al., 2021). The same is true of hEPS cells’ self-renewal and differentiation regulation. WDR36 is a nucleolar protein involved in the maturation of 18S rRNA. The primary structure of the WDR36 protein is similar to that of Utp21 (Footz et al., 2009), an essential nucleolar ribonucleoprotein. It is also essential for early mouse embryonic development, as homozygous WDR36-deficient mouse embryos die before reaching the blastocyst stage (Gallenberger et al., 2011). In addition, WDR5, which has a similar structure to WDR36, can directly interact with the pluripotent transcription factor OCT4 to regulate ESC self-renewal and differentiation (Ang et al., 2011). However, whether WDR36-mediated ribosome biogenesis or other biological events can regulate the self-renewal and differentiation of hEPS cells remain unknown.

In this study, the WDR36 protein localized in the nucleus and cytoplasm in undifferentiated hEPS1 and hEPS2 cells (Figure 1A, Supplementary Figure S1A), which was in line with previous research that WDR36 protein was ubiquitously expressed in the cytoplasm and nucleus of zebrafish embryos. In addition, WDR36 fusions first localized to nucleoli and then became enriched in both the cytoplasm and nucleus of zebrafish embryos as protein levels increased (Skarie and Link, 2008). For WDR36 localized in the cytoplasm, it may be related to the interaction of WDR36 with G protein-coupled receptors (GPCRs). WDR36 acts as a scaffold protein tethering G-protein-coupled receptors, Gαq and PLCβ, in a signaling complex (Cartier et al., 2011; Blankenbach et al., 2020). GPCRs are the largest class of cell surface receptors and therefore mediate a variety of biological processes (Wootten et al., 2018). For example, they are developmental regulators of cell morphology, polarity, and migration (Wettschureck and Offermanns, 2005; Cotton and Claing, 2009). Most importantly, the role of GPCRs in stem cell maintenance is indubitably important, and they contribute to ESCs’ self-renewal, pluripotency, and the maintenance of the clonal morphology (Layden et al., 2010). In addition, GPCRs also regulate the mesoderm differentiation of stem cells through lipid-mediated signaling pathways and Wnt signaling pathways (Kobayashi et al., 2010).

To broaden the effect of WDR36 on hEPS cells, we removed the LCDMY cocktail which was essential for hEPS cell maintenance from the N2B27-LCDMY culture condition and cultured cells for two to three passages. In hEPS1, we observed almost no morphological change on day 3 of P0, but the doomed colonies gradually flattened and collapsed later, whereas in hEPS2, we observed morphological changes as early as day 3 at P0, and the doomed colonies gradually flattened and collapsed afterward. After being exposed to the condition without LCDMY cocktail for one or two passages, all the hEPS cells lost their EPS identity with no OCT4 or WDR36 expression. Moreover, the expression of WDR36 and pluripotent transcription factors decreased, along with the embryonic and extraembryonic lineages differentiation genes being activated (Figures 1E–G and Supplementary Figures S1D,F). In addition, both siRNA-WDR36 and shRNA-WDR36 impaired the self-renewal of hEPS cells (Figures 2C,D and Supplementary Figure S1), reminding a pivotal role of WDR36 in hEPS cells’ self-renewal. Notably, studies have shown that *OCT4* and *NANOG* were significantly downregulated in feeder-free EPS cells, compared with primed ESC, and WDR36 was also downregulated in feeder-free EPS cells, although not significant (Zheng et al., 2021), which was consistent with our results.

Furthermore, the expression of *P53*, *P21*, *MDM2*, and *BAX* increased during hEPS1 cell differentiation, and so they did in WDR36 knockdown hEPS1 cells (Figures 5A–C). Previous studies have shown that WD40 repeat proteins are involved in regulating a variety of cellular functions, such as cell division, cell fate determination, and transmembrane signaling (Li and Roberts, 2001). Disruption of WDR36 in HTM-N cells causes apoptosis and upregulation of *P53*, *P21*, and *BAX* expressions (Gallenberger et al., 2011). The same phenomenon also occurs during embryonic development, that is, the loss of WDR36 expression disrupts nucleolus homeostasis and results in significant upregulation of P53 (Skarie and Link, 2008). Indeed, P53 plays a key role in actively promoting the differentiation of human ESCs. Before ESC differentiation, the expression level of P53 is very low in human ESCs, and HDM2 and TRIM24, two negative regulators of P53, trigger P53 degradation. Active P53 in turn promotes the expression of a cell cycle regulator P21, which slows down human ESC by prolonging the cell cycle gap (G1) phase (Jain et al., 2012; Ghatak et al., 2021). In addition, many studies have shown that P53 has many downstream target genes that play regulatory roles, including some non-coding RNAs, such as miR-34a and miR-145, currently being identified as regulated by P53 (Hermeking, 2007; Jain et al., 2012). Recently, Dox-inducible exogenous expression of P21 in human ESCs was shown to induce cell cycle arrest and substantial human ESC differentiation (Ruiz et al., 2011), further supporting that induced expression of P21 is required for human ESCs to differentiate. Moreover, the ubiquitin ligase MDM2 is best known for balancing the activity of the tumor suppressor P53, but it can also promote adipocyte differentiation in a P53-independent manner (Hallenborg et al., 2016), and hESC differentiation can change the status of active BAX and sensitivity to DNA damage (Dumitru et al., 2012).

Evidence suggests that the P53 family is critical for mesodermal specification during exit from pluripotency in the embryo and culture. Wnt3 and its receptor Fzd1 are direct target genes of the P53 family, and induction of Wnt signaling by P53 is critical for the activation of mesodermal differentiation genes (Wang et al., 2017). In addition, transient WDR5 inhibition can stimulate ESC differentiation toward mesodermal fate *via* P53 as well as allow global chromatin accessibility to the landscape of mesodermal differentiation (Li et al., 2020). In the current study, WDR36 knockdown significantly promoted the differentiation of hEPS cells to the mesoderm lineage (Figures 3,4, Supplementary Figure S5), indicating that WDR36 plays a key role in committing the mesodermal fate of hEPS cells. Therefore, we detected whether P53 signaling inhibition would reverse the effect of WDR36 knockdown on hEPS. Several works have proven that supplementation of PFT-α in the culture medium can effectively inhibit P53 (Garbern et al., 2020; Samarasinghe et al., 2021). In the current study, we treated hEPS1 cells with 2, 4, 6, 8, and 10 µM PFT-α, and the expressions of P53 and P21 were significantly inhibited at 10 μM, without affecting the survival of hEPS1 cells (Supplementary Figures S8A,B). As expected, inhibition of p53 signaling reversed the phenomenon that WDR36 knockdown attenuated self-renewal and enhanced mesoderm differentiation (Supplementary Figures S8C,F).

Collectively, our research showed that WDR36 was downregulated during hEPS differentiation. By constructing hEPS cell lines with Dox-induced WDR36 overexpression and WDR36 silencing, we further found that WDR36 knockdown disrupted self-renewal but promoted differentiation, especially toward the mesodermal lineage. Moreover, P53 signaling inhibition would reverse the effect of WDR36 knockdown on hEPS, in which the pluripotency and differentiation genes and the P53 signaling pathway-related factors were all altered. Taken together, our study provides a new insight into how WDR36 influences hEPS cells’ self-renewal and differentiation.

## Data Availability

The original contributions presented in the study are included in the article/Supplementary Material; further inquiries can be directed to the corresponding author.
