# Supplementary material for: WDR36 Safeguards Self-Renewal and Pluripotency of Human Extended Pluripotent Stem Cells
Source: Front Genet. 2022 Jul 22;13:905395. doi: 10.3389/fgene.2022.905395 (PMC9353684; doi:10.3389/fgene.2022.905395)
Supplement: Supplementary file 1 [file DataSheet1.docx]

Supplementary Material

# Supplementary Figures and Tables

## Supplementary Figures


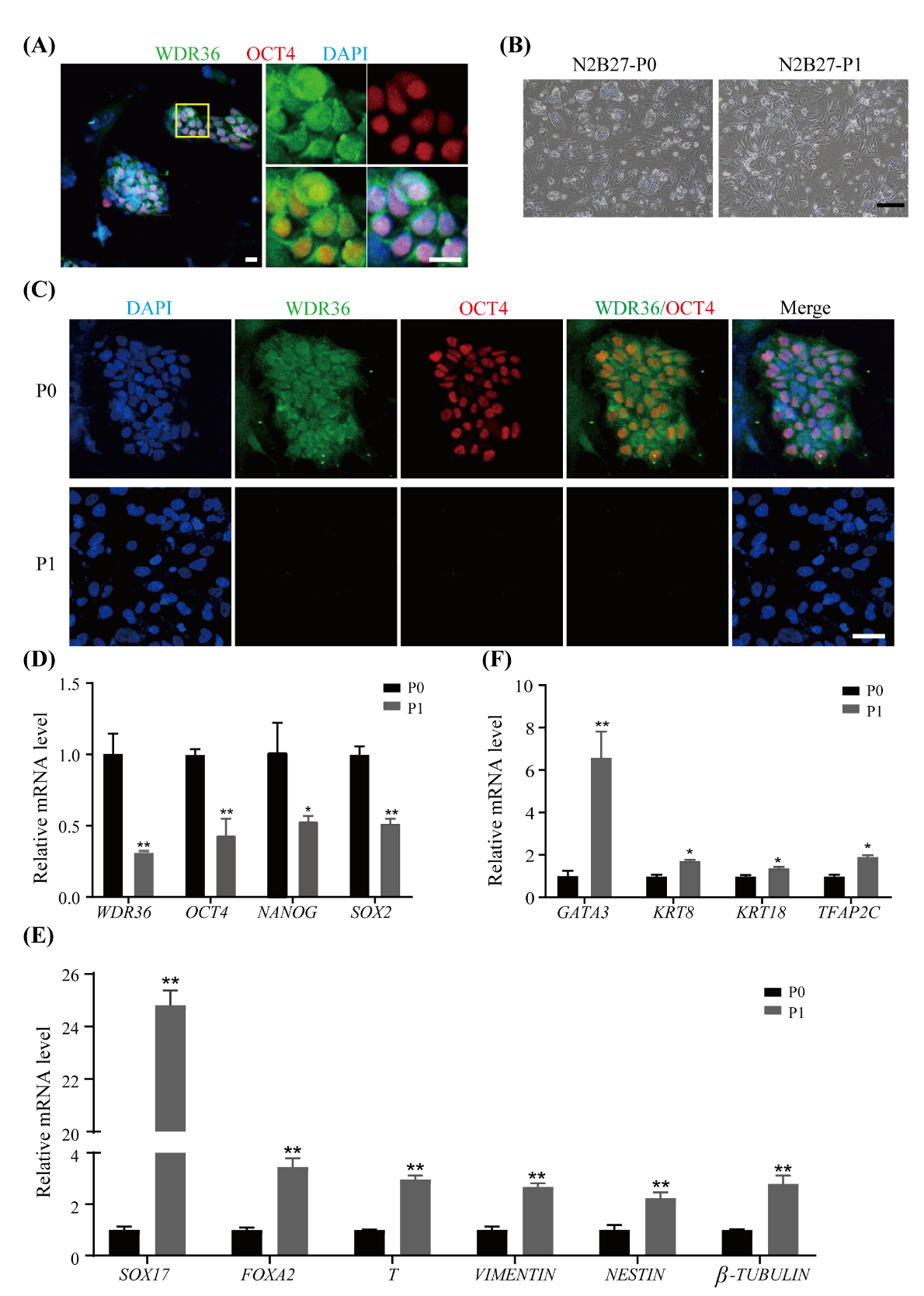


**Supplementary Figure 1.** **Down-expression of WDR36 during hEPS2 cells differentiation.** (A) Expression of WDR36 protein in hEPS2 cells. Bars=20 μm. (B-C) Representative brightfields (B, Bars=200 μm) and immunofluorescent images (C, Bars=50 μm) of hEPS2 cells in the spontaneous differentiation assay at P0 and P1, separately. (D-F) Expression of pluripotent genes (D), embryonic germ layer related genes (E) and extraembryonic differentiation related genes (F) in hEPS2 cells cultured in N2B27 medium at P0 and P1. n=3 experiments; mean ± S.D; two-tailed Student’s t- test. *, 0.01< *p* < 0.05; **, *p* < 0.01; no labeling indicates no statistical significance.


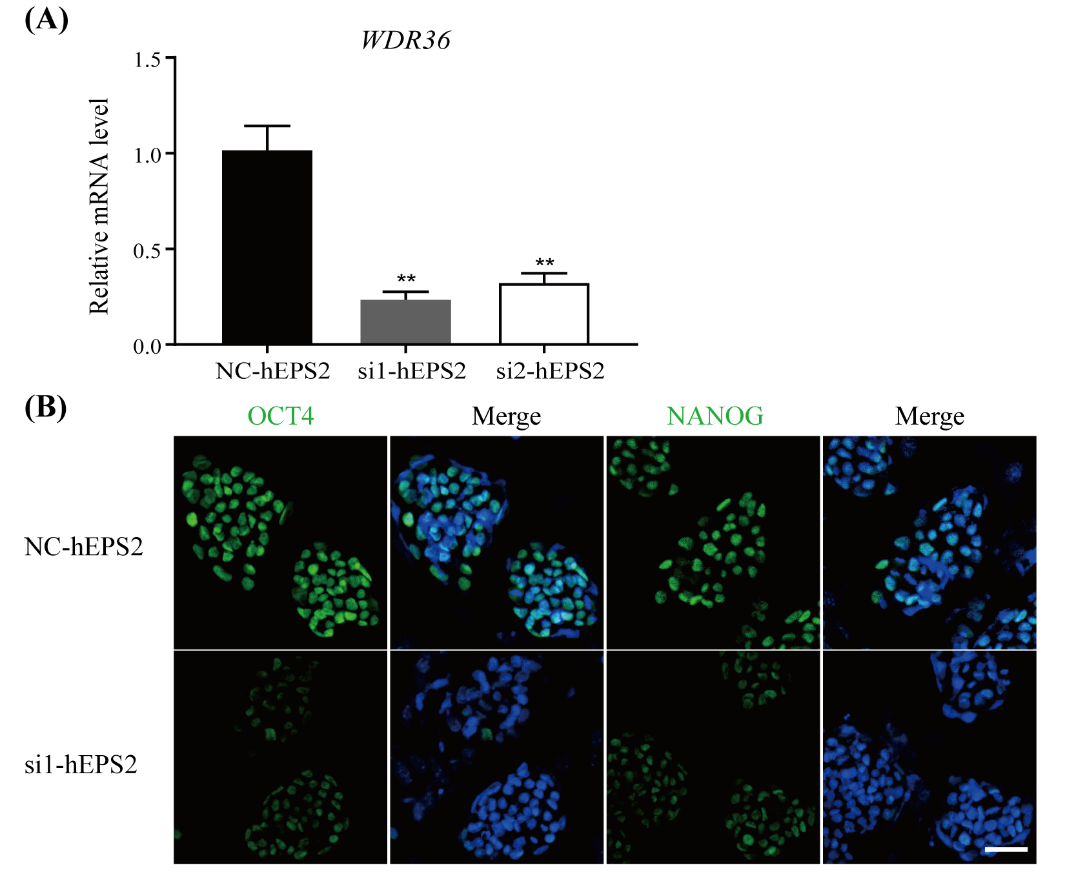


**Supplementary Figure 2. siRNA-WDR36 would impair self-renewal of hEPS2 cells.** (A) WDR36 expression level in hEPS2 cells with WDR36 siRNA was determined using qRT-PCR. n=3 experiments; mean ± S.D; two-tailed Student’s t- test. *, 0.01< *p* < 0.05; **, *p* < 0.01. (B) Immunofluorescent images of OCT4 and NANOG in hEPS2 cells with WDR36 siRNA. Bar =50 μm. NC-hEPS2 and si1-hEPS2: hEPS2 cells transfected with siRNA-scramble and small interference RNA 1 of WDR36 respectively.


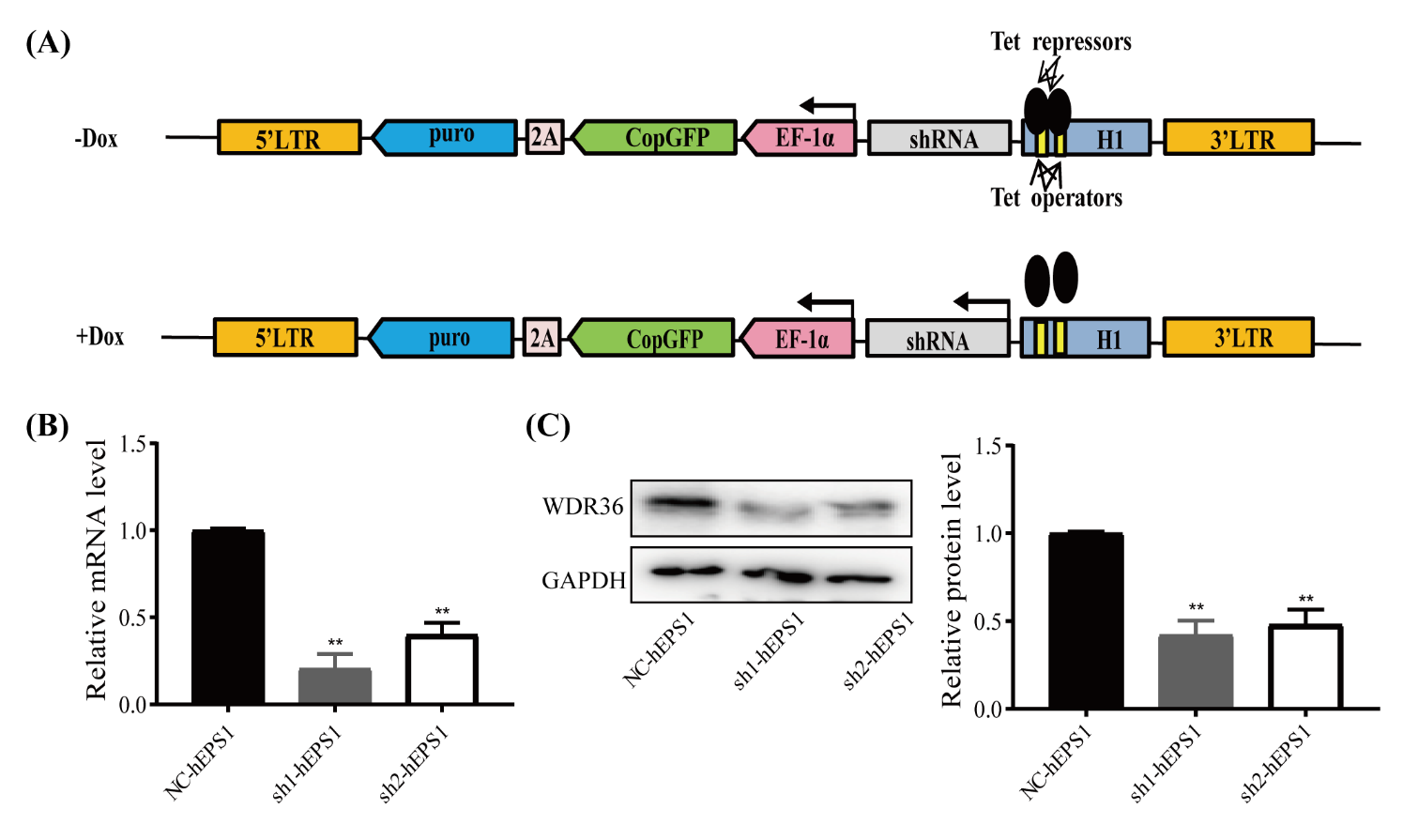


**Supplementary Figure 3. Construction and identification of Dox-induced WDR36-silenced hEPS1 cells.** (A) Schematic diagram depicting the generation of Dox-induced WDR36-silenced hEPS1 cells. (B-C) The mRNA (B) and protein (C) expression levels of *WDR36* following Dox treatment in Dox-induced WDR36-silenced hEPS1 cells. Data are presented as mean ± SD in three independent experiments and student’s t tests are used for statistical analysis. *, 0.01< *p* < 0.05; **, *p* < 0.01. NC-hEPS1, sh1-hEPS1 and sh2-hEPS1: hEPS1 cells transfected with shRNA-scramble and short hairpin RNA 1, 2 of *WDR36* respectively.


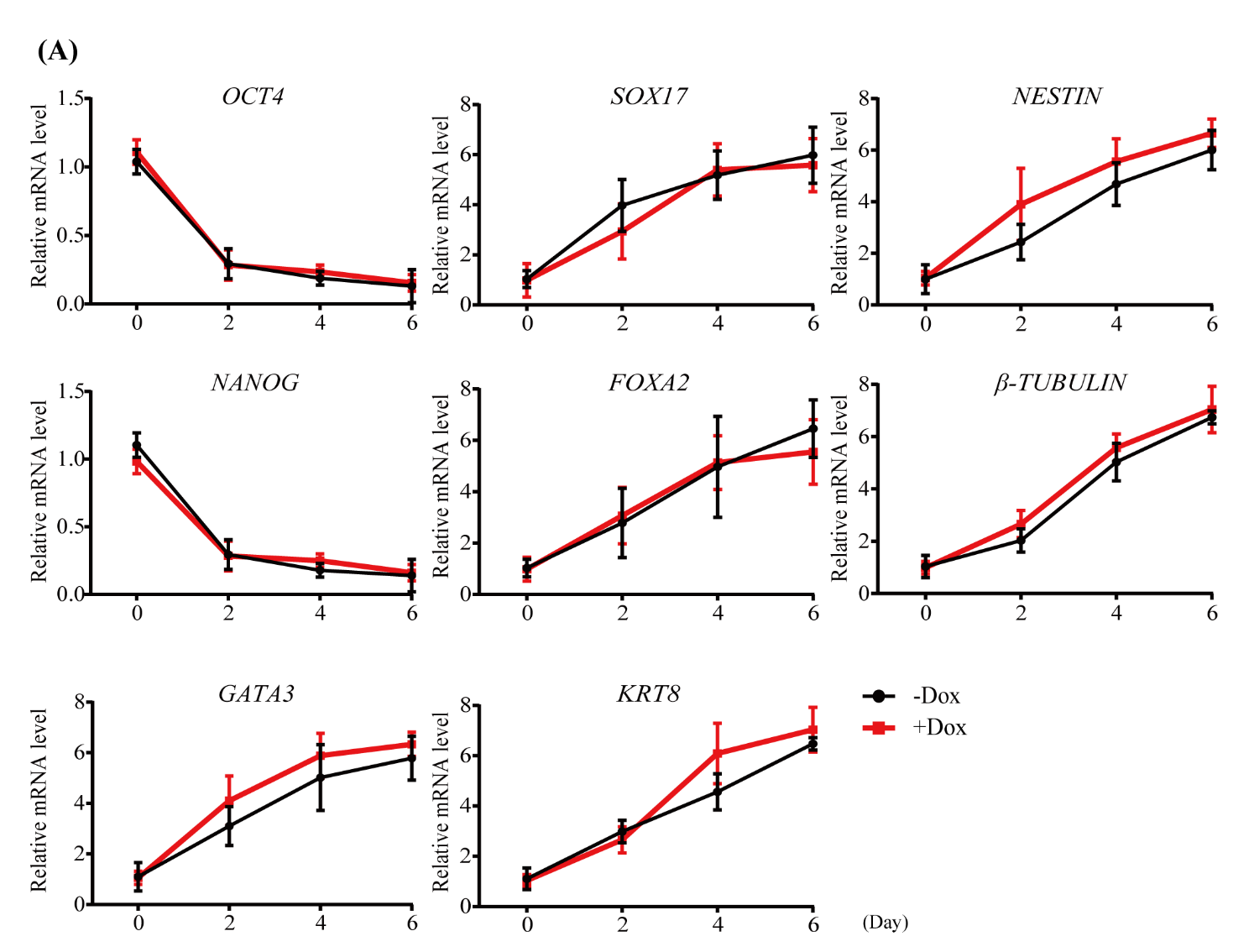


**Supplementary Figure 4. Dox-induced knockdown of WDR36 had no obvious effects on ectodermal, endodermal and extraembryonic differentiation of hEPS1 cells in an EB assay.** (A) The mRNA expression levels of pluripotent, endodermal, ectodermal and extraembryonic differentiated marker genes in the sh1-hEPS1-derived EBs in the present or absence of Dox. Data are presented as mean ± SD in three independent experiments and student’s t tests are used for statistical analysis. *, 0.01< *p* < 0.05; **, *p* < 0.01; no labeling indicates no statistical significance.

**
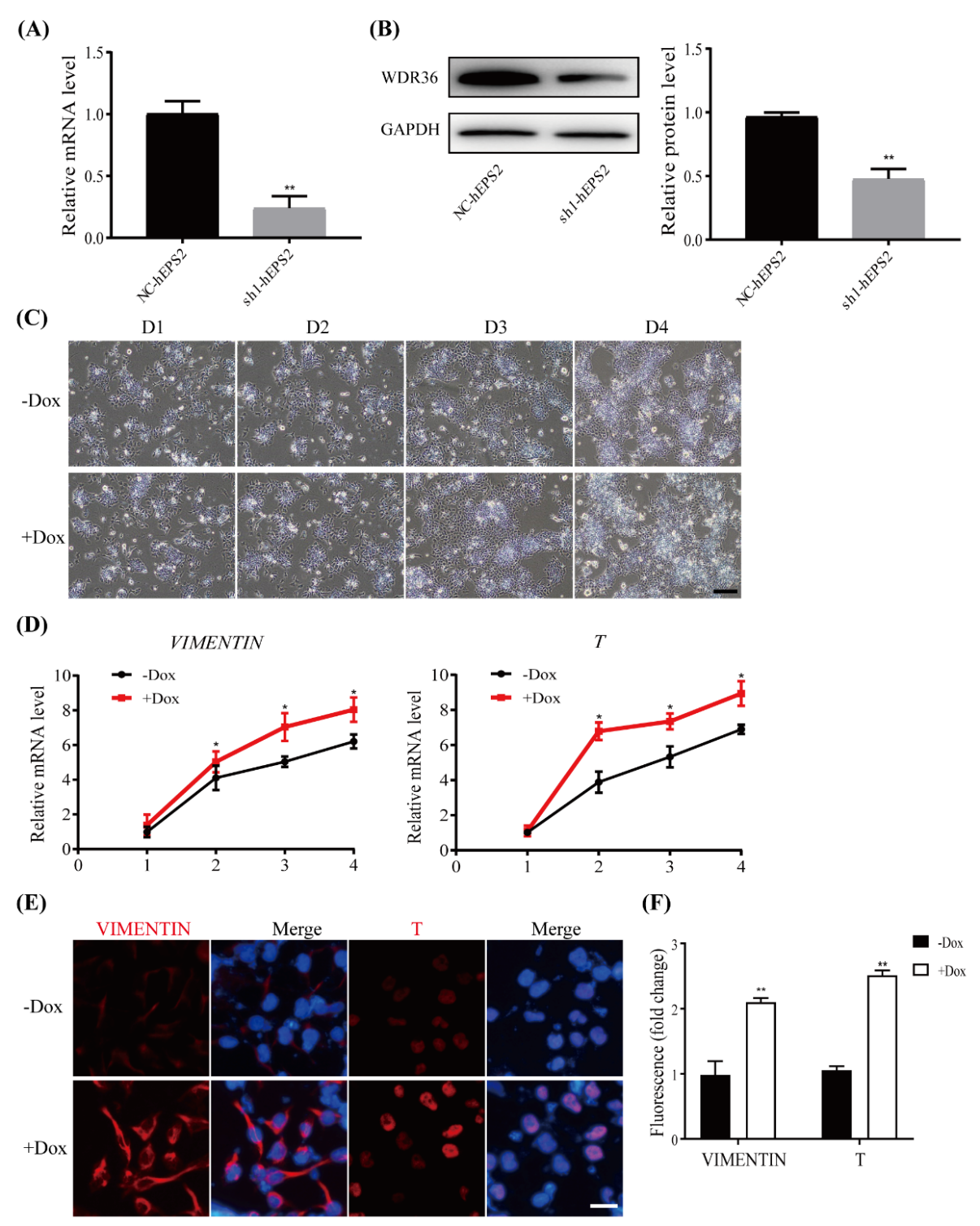
**

**Supplementary Figure 5. WDR36 knockdown promoted mesodermal differentiation potential of hEPS2 cells in a mesodermal committed differentiation assay.** (A-B) The mRNA (A) and protein (B) expression levels of WDR36 following Dox treatment in Dox-induced WDR36-silenced hEPS2 cells. (C) Representative images of Dox-induced shWDR36 hEPS2 cells in mesoderm differentiation medium with or without Dox. Bar=200 μm. (D-E) mRNA (D) and protein expression (E) of T and VIMENTIN in Dox-induced shWDR36 hEPS2 cells treated with or without Dox. (F) Quantification intensity levels for immunofluorescence of T and VIMENTIN in Dox-induced shWDR36 hEPS2 cells treated with or without Dox. Bar=50 μm. n=3 experiments; mean ± S.D; two-tailed Student’s t test. *, 0.01< *p* < 0.05; **, *p* < 0.01; no labeling indicates no statistical significance.

**
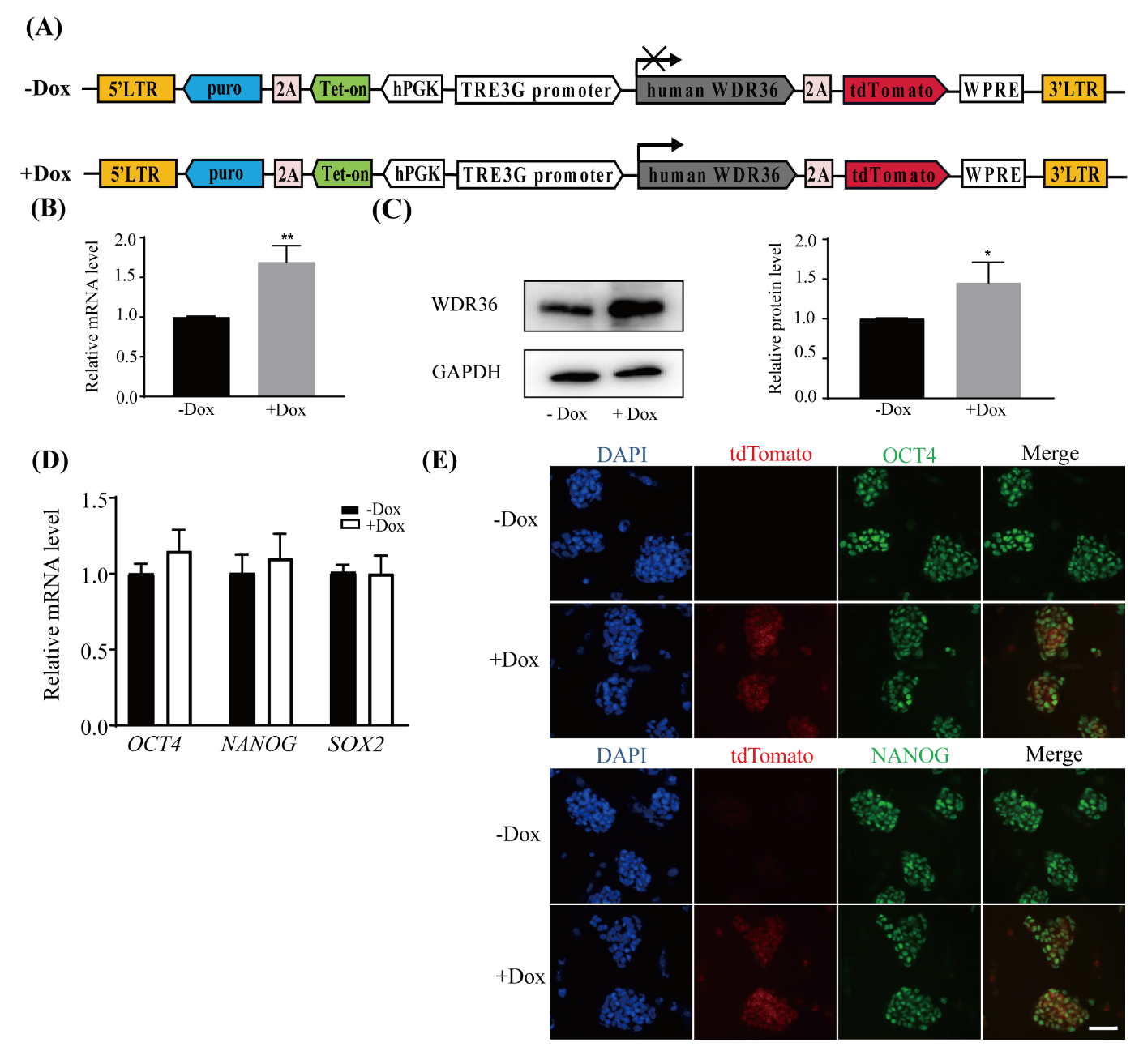
Supplementary Figure 6. Dox-induced overexpression of WDR36 hardly affected self-renewal in hEPS1 cells.** (A) Schematic diagram depicting the generation of Dox-induced WDR36-overexpressing hEPS1 cells. (B-C) The mRNA (B) and protein (C) expression levels of WDR36 following Dox treatment. (D) Expression of *OCT4*, *NANOG* and *SOX2* in Dox-induced WDR36 overexpression hEPS1 cells treated with or without Dox. (E) Immunofluorescent images of Dox-induced WDR36 overexpression hEPS1 cells treated with or without Dox. Bar =50 μm. Data are presented as mean ± SD in three independent experiments and student’s t tests are used for statistical analysis. *, 0.01< *p* < 0.05; **, *p* < 0.01; no labeling indicates no statistical significance.


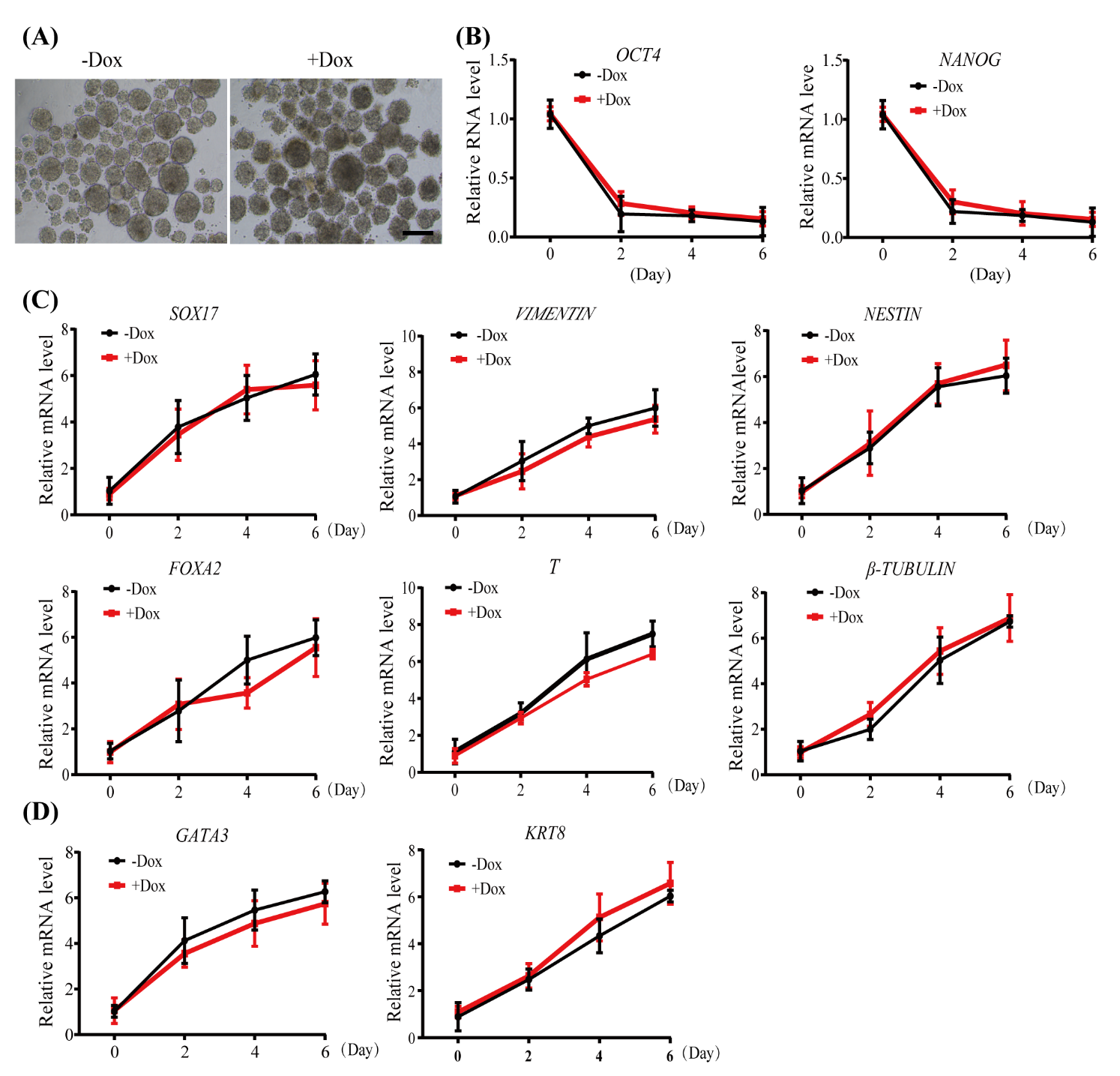


**Supplementary Figure 7. Dox-induced overexpression of WDR36 had no pronounced effect on ectodermal, endodermal and extraembryonic differentiation of hEPS1 cells.** (A) WDR36 overexpression hEPS1 cells formed EBs in EB medium. On day 7, representative EBs were collected and observed under the microscope (Bar=200 μm). (B-D) The mRNA expression levels of pluripotent, endodermal, ectodermal and extraembryonic differentiated marker genes in the Dox-induced WDR36 overexpression hEPS1 cells. Data are presented as mean ± SD in three independent experiments and student’s t tests are used for statistical analysis. *, 0.01< *p* < 0.05; **, *p* < 0.01; no labeling indicates no statistical significance.


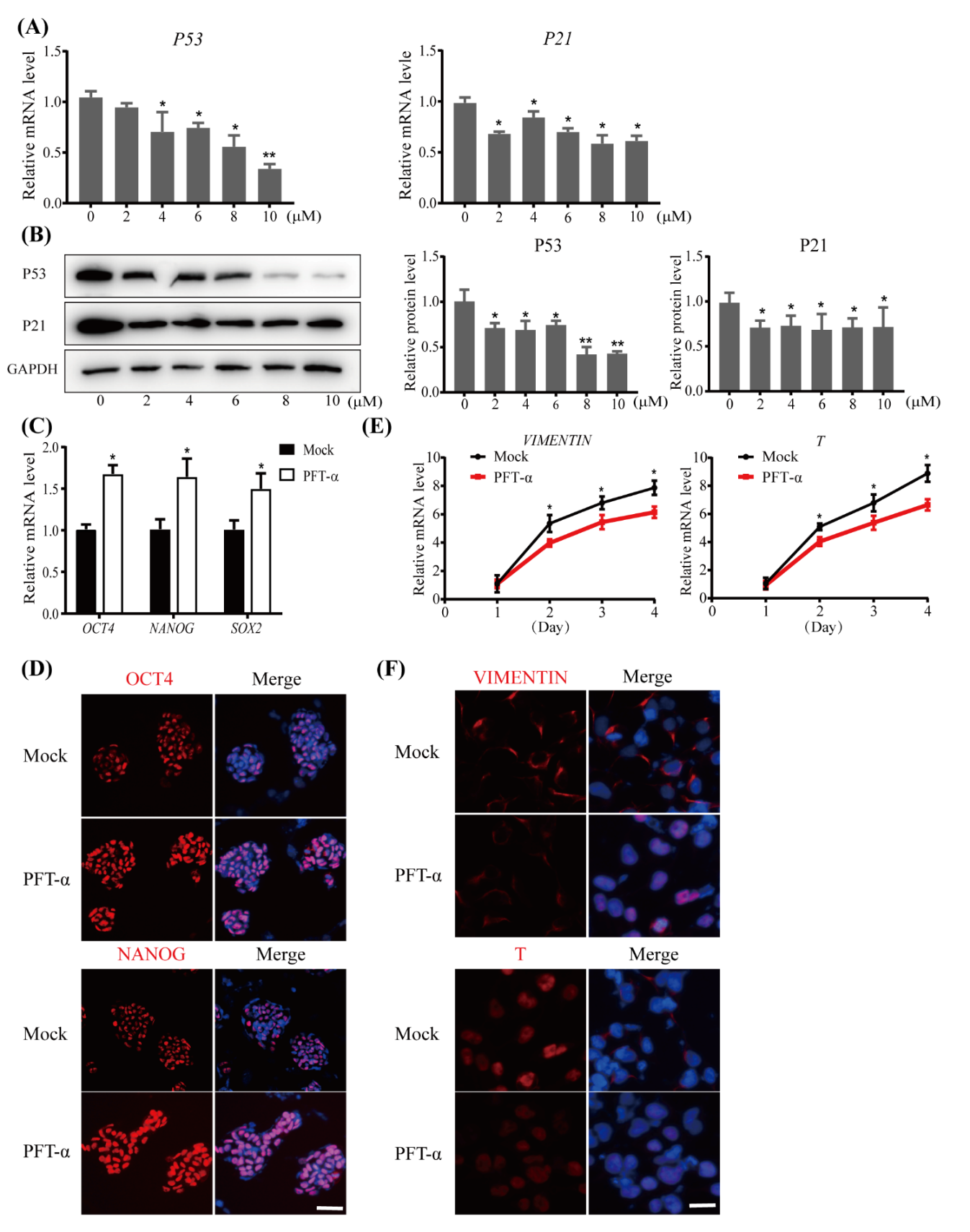


**Supplementary Figure 8. PFT-α promoted self-renewal of hEPS1 cells *in vitro*.** (A-B) The mRNA and protein expression levels of *P53* and *P21* following PFT-α treatment. (C-D) mRNA (C) and protein expression (D) of OCT4, NANOG and SOX2 in hEPS1 cells treated with or without PFT-α. (E-F) mRNA (E) and protein expression (F) of mesodermal marker genes in hEPS1 cells treated with or without PFT-α. Bars=50 μm. PFT-α was used at 10 μM. Data are presented as mean ± SD in three independent experiments and student’s t tests are used for statistical analysis. *, 0.01< *p* < 0.05; **, *p* < 0.01; no labeling indicates no statistical significance.

## Supplementary tables

**Supplement Table 1. Details of antibodies used for IF and WB in this study.**

| Antibodies name | Cat NO. | Source | Dilutions used in IF | Dilutions used in WB |
| --- | --- | --- | --- | --- |
| Rabbit anti-WDR36 polyclonal antibody | ab73548 | Abcam | 1:200 | 1:500 |
| Oct-3/4 (C-10) antibody | sc-5279 | Santa Cruz | 1:500 | 1:500 |
| NANOG Polyclonal Antibody | 14295-1-AP | Proteintech | 1:500 | 1:1000 |
| SOX2 Polyclonal Antibody | 11064-1-AP | Proteintech | 1:500 | 1:1000 |
| Human/Mouse Brachyury Antibody | AF2085 | R&D Systems | 1:500 | 1:1000 |
| Mouse monoclonal Anti-Vimentin antibody | ab20346 | Abcam | 1:500 | 1:1000 |
| Human SOX17 Antibody | AF1924 | R&D Systems | 1:500 | 1:1000 |
| Nestin Antibody (10c2) | sc-23927 | Santa Cruz | 1:500 | 1:1000 |
| P53 Polyclonal Antibody | 10442-1-AP | Proteintech | - | 1:1000 |
| P21 Polyclonal Antibody | 10355-1-AP | Proteintech | - | 1:1000 |
| GAPDH antibody | 60004-1-Ig | Proteintech | − | 1:8000 |
| HRP-conjugated Affinipure Goat Anti- Rabbit | SA00001-2 | Proteintech | − | 1:4000 |
| HRP-conjugated Affinipure Goat Anti-Mouse | SA00001-1 | Proteintech | − | 1:4000 |
| Donkey anti-Mouse IgG, Alexa Fluor 488 | A21202 | Thermo Fisher scientific | 1:500 | − |
| Donkey anti-Rabbit IgG, Alexa Fluor 488 | A21206 | Thermo Fisher scientific | 1:500 | − |
| Donkey anti-Mouse IgG, Alexa Fluor 555 | A31570 | Thermo Fisher scientific | 1:500 | − |
| Donkey anti-Rabbit IgG, Alexa Fluor 555 | A31572 | Thermo Fisher scientific | 1:500 | − |

IF: Immunofluorescence; WB: Western blot; (−), absent.

**Supplement Table 2. Details of primer sequences, expected product sizes and Genbank accession numbers of genes used for qPCR.**

| Gene name | Primer sequence (5′- 3′) | Product length (bp) |
| --- | --- | --- |
| *WDR36* | F: TGGCAAGGATCAAGCTCACAA  R: AAGTCATCCAAGGCGAGTCC | 210 |
| *OCT4* | F: CTTGAATCCCGAATGGAAAGGG  R: GTGTATATCCCAGGGTGATCCTC | 262 |
| *NANOG* | F: CAGCCCCGATTCTTCCACCAGTCCC  R: CGGAAGATTCCCAGTCGGGTTCACC | 217 |
| *SOX2* | F: GCCGAGTGGAAACTTTTGTCG  R: GGCAGCGTGTACTTATCCTTCT | 155 |
| *T* | F: GATGATCGTGACCAAGAACGG  R: CCACGAAGTCCAGCAGGAA | 104 |
| *Vimentin* | F: AATGACCGCTTCGCCAACTA  R: TCCTCCTGCAATTTCTCCCG | 245 |
| *SOX17* | F: GCATGACTCCGGTGTGAATCT  R: TCACACGTCAGGATAGTTGCAGT | 103 |
| *FOXA2* | F: CATGTTGCTCACGGAGGAGT  R: TTTAAACTGCCATGCACTCG | 122 |
| *Nestin* | F: TCAGCTTTCAGGACCCCAAG  R: GGTGTCTCAAGGGTAGCAGG | 130 |
| *β-tublin* | F: ATCAGCAAGGTGCGTGAGGAG  R: TCGTTGTCGATGCAGTAGGTC | 161 |
| *GATA3* | F: AAGGCATCCAGACCAGAAACCG  R: AGCATCGAGCAGGGCTCTAACC | 273 |
| *KRT8* | F: ACCCTCAACAACAAGTTTGCCTCC  R: TCCACTTGGTCTCCAGCATCTTGT | 82 |
| *KRT18* | F: ACACAGTCTGCTGAGGTTGGAG  R: TGCTCCATCTGTAGGGCGTAG | 161 |
| *TFAP2C* | F: CGCTCATGTGACTCTCCTGACATCC  R: TGGGCCGCCAATAGCATGTTCT | 180 |
| *p53* | F: CCAGAAAACCTACCAGGGCA  R: GAATGCAAGAAGCCCAGACG | 51 |
| *p21* | F: GGCCCAGTGGACAGCGAGCA  R: CCCAGGCGAAGTCACCCTCC | 130 |
| *MDM2* | F: GGCTTTGATGTTCCTGATTG  R: CTTTGTCTTGGGTTTCTTCC | 202 |
| *BAX* | F: TGGAAGAAGATGGGCTGAG  R: GTGTCCCGAAGGAGGTTTA | 179 |
| *GAPDH* | F: TCGACAGTCAGCCGCATCTTCTTT  R: ACCAAATCCGTTGACTCCGACCTT | 186 |

**Supplement Table 3. Primer sequences of siRNA and shRNA in this study.**

| Name | Sequence (5′- 3′) |
| --- | --- |
| siRNA-scramble | F: UUCUCCGAACGUGUCACGUTT  R: ACGUGACACGUUCGGAGAATT |
| siRNA1 | F: GCCGUGGAUGUUGUUGCUAUUTT  R: AAUAGCAACAACAUCCACGGCTT |
| siRNA2 | F: CAGGCAUACCUUGCAUUGUUUTT  R: AAACAAUGCAAGGUATGCCUGTT |
| shRNA-scramble | GTTCTCCGAACGTGTCACGT |
| shRNA1 | CCGGGCCGTGGATGTTGTTGCTATTCTCGAGAATAGCAACAACATCCACGGCTTTTTT |
| shRNA2 | CCGGCCTTGGATGACTTCTCCATTACTCGAGTAATGGAGAAGTCATCCAAGGTTTTTT |
